# Decision Support and the Effectiveness of Web-based Delivery and Information Tailoring for Bowel Cancer Screening: An Exploratory Study

**DOI:** 10.2196/resprot.2135

**Published:** 2012-09-26

**Authors:** Ingrid H Flight, Carlene J Wilson, Ian T Zajac, Elizabeth Hart, Jane A McGillivray

**Affiliations:** 1 Preventative Health Research FlagshipCommonwealth Scientific and Industrial Research Organisation (CSIRO)Adelaide BCAustralia; 2 School of PsychologyDeakin UniversityBurwood, Vic 3125Australia; 3 Flinders Centre for Cancer Prevention and ControlFlinders UniversityDaw Park, SA 5042Australia; 4 Cancer Council South AustraliaEastwood, SA 5063Australia

**Keywords:** Colorectal cancer, mass screening, multimedia, communication, decision support techniques

## Abstract

**Background:**

Colorectal cancer (CRC) is the third most commonly diagnosed cancer in males and the second in females throughout the developed world. Population screening using fecal occult blood tests (FOBTs) facilitates early detection and greater chance of survival, but participation rates are low. We developed a Web-based decision tool to provide information tailored to an individual’s decision stage for CRC screening and attitude toward screening utilizing the Preventive Health Model (PHM) and Precaution Adoption Process Model (PAPM) as theoretical frameworks for screening behavior. We describe the practical steps employed in the tool’s design and the subsequent conduct of an exploratory study.

**Objective:**

To design a decision tool for CRC screening and conduct an exploratory study among average-risk men and women to (1) test the impact of message type (tailored vs non-tailored) and message delivery modality (Web-based vs paper-based) on attitudes toward screening and screening uptake, and (2) investigate the acceptability of the decision tool and relevance of materials.

**Methods:**

Participants (n = 100), recruited from a population sample of men and women aged 50-76 residing in urban Adelaide, Australia, were randomly assigned to a control group or one of 4 interventions: (1) Web-based and tailored information, (2) paper-based and tailored information, (3) Web-based and non-tailored (generic) information, or (4) paper-based and non-tailored information. Participation was augmented by snowball recruitment (n = 19). Questionnaires based on PHM variables were administered pre- and post-intervention. Participants were given the opportunity to request an FOBT. Following the intervention, participants discussed the acceptability of the tool.

**Results:**

Full data were available for 87.4% (104/119) of participants. Post-intervention, perceived susceptibility scores for individuals receiving tailored information increased from mean 10.6 (SD 2.1) to mean 11.8 (SD 2.2). Scores on self-efficacy increased in the tailored group from mean 11.7 (SD 2.0) to mean 12.6 (SD 1.8). There were significant time x modality x message effects for social influence and salience and coherence, reflecting an increase in these scores for tailored Web-based participants only; social influence scores increased from mean 11.7 (SD 2.6) to mean 14.9 (SD 2.3), and salience and coherence scores increased from mean 16.0 (SD 2.2) to mean 17.7 (SD 2.1). There was no greater influence of modality or message type on movement toward a decision to screen or screening uptake, indicating that neither tailored messages nor a Web modality had superior effect. Overall, participants regarded tailored messages positively, but thought that the Web tool lacked “media richness.”

**Conclusions:**

This exploratory study confirms that tailoring on PHM predictors of CRC screening has the potential to positively address attitudes toward screening. However, tailoring on these variables did not result in significantly increased screening uptake. Future research should consider other possible psychosocial influences. Mode of delivery did not affect outcomes, but as a delivery medium, the Web has economic and logistical advantages over paper.

## Introduction

Colorectal cancer (CRC) is the third most commonly diagnosed cancer in males and the second in females throughout the developed world [1]. Population screening using a fecal occult blood test (FOBT), or the second-generation fecal immunochemical test (FIT), facilitates the detection of CRC at its early stages by detecting invisible (occult) traces of blood in the feces. A recent systematic review concluded that appropriate population utilization of FOBT screening is likely to reduce death from CRC by about 1 in 6 deaths [2]. This possibility has resulted in the implementation of national pilot or population screening programs in a number of countries [3]. Effectiveness of these programs depends upon yield and participation rates—in Australia, estimates of participation in screening for colorectal cancer have been low [4]. The National Bowel Cancer Screening Program (NBCSP), which provides people turning 50, 55, and 60 years with a free FOBT, had a total participation rate over three years (June 2008 to June 2011) of 38.4% of the eligible population [5].

This paper describes the theoretical framework and practical steps we employed to design a Web-based, tailored decision tool for CRC screening, and to conduct an exploratory study to test its impact on screening attitudes and uptake prior to the design and conduct of a larger randomized trial. We examined uptake of FOBT only (versus colonoscopy or in addition to colonoscopy) because unlike other countries, such as the United States, usual CRC screening practice in Australia is by FOBT followed by colonoscopy for those with a positive result—colonoscopy is regarded as a diagnostic test rather than a screening test. We also sought to place the study in the context of the NBCSP approach, which is to encourage population-based screening using FOBT for those who do not have any obvious symptoms of bowel cancer.

### Theoretical Framework

Two classes of behavioral theory are relevant to understanding uptake of new health behavior. “Stage of change” or “readiness to act” models, such as the Precaution Adoption Process Model (PAPM) [6], focus on an individual’s commitment to act. The PAPM characterizes movement toward commitment as (1) unaware of the issue, (2) heard of the issue but unconcerned, (3) considering action, (4) deciding against action, or (5) deciding to act. By contrast, “continuum” theories, such as the Preventive Health Model (PHM) [7], focus on psychosocial predictors of intention to act and on predictors of behavior. The PHM approach identifies 5 variables that affect the likelihood to act. In the context of cancer screening, these are (1) salience and coherence of the screening behavior (the perception that performing cancer screening is consistent with beliefs about how to protect and maintain health); (2) perceived susceptibility (perceptions of personal risk for developing colorectal cancer or polyps); (3) response efficacy (the belief that utilizing an FOBT will be effective in reducing disease threat); (4) cancer worries (concerns about negative consequences of undertaking cancer screening); and (5) social influence (extent to which an individual believes that those who they interact with, and whose opinions they value, support FOBT use). Research indicates that these factors are associated with the decision to screen in the United States [8], Canada [9], and Australia [10]. Two other constructs—self-efficacy and fecal aversion—are important in the context of this study. Self-efficacy, in this case the confidence to utilize the FOBT, is a significant predictor of health-related intentions [11] and behaviors [12], and fecal aversion has been demonstrated to be a predictor of FOBT uptake [13].

Research has shown that programs designed to improve screening uptake are enhanced considerably when stage theories and continuum theories are utilized together. Groups of people at a specific stage of thinking about CRC screening (PAPM) can be distinguished from people at a different stage in terms of their responses to the variables included in the PHM [14,15]. Thus, utilization of both the PAPM and PHM can enable the provision of messages that are “tailored” to individual responses with the aim of moving people to a “better” screening decision stage.

### Tailored Communication

Tailored health promotion materials are “...any combination of information and behavior change strategies intended to reach one specific person, based on characteristics that are unique to that person, related to the outcome of interest, and derived from an individual assessment” [16]. Tailored print communication is better remembered, read, and perceived as more relevant than non-tailored materials [17]. However, a recent systematic review based on two trials, found no evidence of tailored interventions on CRC screening uptake [18], although the researchers found evidence for a beneficial effect of tailored information on perception of cancer risk and knowledge of cancer. Thus, further research is required to better understand the “ingredients” of tailoring approaches on CRC screening rates.

### Web Delivery of Information

Paper-based delivery of non-tailored screening messages has improved FOBT uptake [19,20] and tailoring may have the capacity to achieve further incremental improvements in uptake rates. A meta-analysis showed that computerized tailoring (feedback composed by means of computer algorithms) demonstrated improved outcomes in terms of health behaviors compared to controls [21]. Using computers to construct tailored messages can facilitate the creation of finer-grained tailored materials; without them, tailoring has been limited to few variables because of constraints on the logistical practicality of creating a comprehensive library of messages and manipulating them to simultaneously address multiple variables [22]. Additionally, delivery via online communication channels may increase the feasibility of this individualized approach to communication through channels such as interactive feedback and simplicity of navigation to personally relevant materials. For example, a meta-analysis comparing Web-based and non-Web-based information interventions has shown enhanced outcomes among individuals using Web-based interventions, particularly in the areas of knowledge and targeted behavior change [23].

In light of the above considerations, we conducted an exploratory study to investigate whether information tailored to an individual’s current decision stage for screening based on PHM variables would have a greater influence on the readiness of invitees to be screened compared with non-tailored generic information. We also sought to disentangle outcomes and investigate whether it was perceived personal relevance (through tailoring) of information, simplicity of navigation (through Web-based delivery) to access personally relevant material, or both factors that had the greater effect. The primary outcomes were (1) change in PHM scores, (2) change in PAPM decision stage, (3) intention to screen as measured by a request for an FOBT, and (4) actual uptake of screening. We were also interested in participants’ opinions of the acceptability and relevance of the materials. We aimed to use results from this exploratory study to inform the design and conduct of a larger, nationwide randomized controlled trial.

## Methods

### Study Design

A 2 × 2 × 2 mixed model factorial design examined the influence of message type (tailored vs non-tailored) and message modality (paper vs Web) on predictors of screening (PHM variables) and stage of readiness to screen (PAPM stage) measured before and after receipt of screening messages. We planned to contact at least 750 participants to achieve a sample size deemed practical for an exploratory study (n = 125).

#### Pre-intervention (Time 1)

A baseline survey was taken 2 weeks before intervention. The variables measured were demographics, decision stage for screening, decisional conflict, PHM, and self-efficacy and fecal aversion variables.

#### Intervention

Intervention group participants received one of 4 interventions: (1) Web-based and tailored information, (2) paper-based and tailored information, (3) Web-based and non-tailored (generic) information, or (4) paper-based and non-tailored information. The factorial design is illustrated in Table 1.

**Table 1:** Information supplied to intervention group participants based on message type and modality.

Modality	Message	
	Tailored	Non-tailored
Web	PHM feedback and educational material	Generic information and educational material
Paper	PHM feedback and educational material	Generic information and educational material

#### Post-intervention (Time 2)

An endpoint survey and interviews immediately followed intervention. Variables measured were decision stage for screening, decisional conflict, relevance and navigability of the information, PHM, and self-efficacy and fecal aversion variables.

### Participant Recruitment

A random subsample of 756 potential invitees aged 50-76, residing in four urban Adelaide areas, were selected (with permission) from a larger sample provided by the Australian Electoral Commission for a related study. Prior to extracting this subsample, telephone contact numbers were obtained by comparing names and addresses against information contained in the electronic White Pages telephone directory. Those persons for whom telephone contact details were not indicated were excluded, as were those whose postal code indicated they lived more than one hours’ travel time from the Commonwealth Scientific and Industrial Research Organisation **(**CSIRO) laboratories. The remaining names were randomized into 4 intervention groups and 1 control group by a researcher not directly connected with the study using a computer-generated random number sequence (Microsoft Office Excel 2003).

### Study Conduct

The trial commenced in August 2007 and proceeded through a number of phases.

#### Phase 1

A notification letter describing the study was mailed to potential participants. They were advised that they were eligible to participate if they were aged 50-76 and ineligible if they were having regular CRC screening or had ever been diagnosed with colorectal cancer or bowel polyps. Those allocated to the intervention groups were also required to have experience using a computer to search the Web and to be willing to attend the CSIRO laboratory.

#### Phase 2

Two weeks (+/- 48 hours) following the notification letter, attempts were made (maximum 3) to telephone individuals and recruit them to the study. A computer-assisted telephone interview (CATI) format was used to collect interview responses (Microsoft Office Access 2003). Informed consent was formally requested and recorded before commencement of the CATI. Participants answered baseline survey questions to collect background demographics and responses to other variables as described previously. Appointments were made with all but the control group participants to visit the CSIRO laboratory and review materials concerned with CRC screening.

#### Phase 3

Two weeks (+/- 48 hours) later, participants attended CSIRO. They were presented with Web-based or paper-based CRC educational content, as allocated by random sampling, to work through as they wished. Additionally, one Web group and one paper group received messages tailored according to their responses to the baseline survey. The non-tailored Web and paper groups received generic information. Participants in each group were aware that the intervention might be Web- or paper-based, but those who received tailored messages were blinded to the fact that others received only generic messages. The process of developing the tailored messages and educational content is described in the Materials section. Participants were also given the opportunity to request an FOBT through either a Web link or a paper form according to intervention group.

#### Phase 4

Immediately following the intervention, participants were asked to complete an endpoint survey that remeasured the same variables as in the baseline survey. Control group participants were telephoned at this point (ie, approximately 2 weeks following the baseline survey) and they completed the endpoint survey through a CATI. Control group respondents whose PAPM stage was “ready to act” were regarded as having requested an FOBT kit. Those who were not ready to act were sent a letter inviting them to contact the researchers should they subsequently wish to receive an FOBT.

#### Phase 5

All intervention group participants completed an additional questionnaire and participated in a discussion group immediately following completion of the endpoint survey. Both the questionnaire and the discussion explored perceptions of the relevance of the information presented, ease of navigation through the materials, and satisfaction with the information they had gained. The interviews were conducted individually or as a group, depending on the number of people attending the session. The questionnaire analyses are not included here because they have been reported in a separate paper with respect to an enhanced version of the decision aid [24].

#### Phase 6

One day after the intervention, an FOBT was mailed to those who requested one. The remaining participants were sent a letter inviting them to contact the researchers should they subsequently wish to receive an FOBT. Receipt of completed tests was recorded by a processing center and de-identified with aggregate participation data relayed to the researchers. People who did not return their test after 6 weeks were sent a reminder letter. Those who did not return their test after 12 weeks were regarded as having not screened.

### Materials

An overview of the materials developed for the study and their presentation is described subsequently, followed by specific details of the various components.

#### Tailored Intervention

Materials for the tailored group were a message library consisting of messages tailored to an individual user’s decision stage for screening and responses to PHM, self-efficacy, and fecal aversion variables, and generic educational content based on the NBCSP consumer information booklet. The booklet provided generalized risk information (ie, > 50 years, certain bowel conditions, and family history).

#### Non-tailored Intervention

Materials for the non-tailored group were a series of generic messages addressing susceptibility, response efficacy, social influence, self-efficacy and fecal aversion, and generic educational content as described above.

#### Web Group

Educational content was provided in hyperlink format with discrete sections. Web pages were clean and used plain language with bulleted and numbered lists, generous white space and line spacing, and large navigation indicators. Users had the ability to increase font size and to change contrast. A Web link provided the ability to order an FOBT.

#### Paper Group

Educational content was provided in booklet form with discrete sections prefaced with a table of contents. Pages were clean and used plain language with bulleted and numbered lists, and generous white space and line spacing. A self-complete form provided the ability to request an FOBT.

### Questionnaire

A series of statements based on PHM [8,7], self-efficacy, and fecal aversion variables were prepared (Table 2). Self-efficacy was measured using 3 statements derived from previous research regarding FOBT use [7]. Fecal aversion was measured using 3 statements derived from previous research [13]. All scales had acceptable internal consistency as measured by Cronbach alpha (Table 2). All responses were measured on a 5-point Likert scale ranging from “strongly disagree” to “strongly agree.”

**Table 2:** Preventive health model (PHM), self-efficacy, and fecal aversion statements.

Factor		Cronbach alpha	Statements
**PHM** **^a^**			
	Salience and coherence	.73	Colorectal cancer screening makes sense to me.
			Having colorectal cancer screening is an important thing for me to do.^b^
			Having colorectal cancer screening can help to protect my health.
			I will be just as healthy if I avoid having colorectal cancer screening.^c^
	Social influence	.62	I want to do what members of my immediate family think I should do about colorectal cancer screening.
			Members of my immediate family think I should have colorectal cancer screening.^b^
			My doctor or health professional thinks I should have colorectal cancer screening.^b^
			I want to do what my doctor or health professional thinks I should do about colorectal cancer screening.
	Cancer worries	.80	I am afraid of having an abnormal colorectal cancer screening test result.
			I am worried that colorectal cancer screening will show that I have colorectal cancer or polyps.
	Perceived susceptibility	.65	The chance that I might develop colorectal cancer is high.
			Compared with other persons my age, I am at lower risk for colorectal cancer.^c^
			It is very likely that I will develop colorectal cancer or polyps.
			The chances that I will develop colorectal polyps are high.^b^
	Response efficacy	.59	When colorectal polyps are found and removed, colorectal cancer can be prevented.^b^
			When colorectal cancer is found early, it can be cured.
Self-efficacy		.75	I think that doing the test would be easy for me.^b^
			Finding time to do the test would be difficult for me.^c^
			Completing the test correctly would be easy for me.
Fecal aversion		.71	Collecting feces for the purpose of bowel cancer screening is unhygienic.^c^
			Collecting feces for the purpose of bowel cancer screening is distasteful.^b,c^
			Giving a sample of feces to another person is embarrassing.^c^

^a ^Preventive Health Model (PHM) construct descriptions and survey items reproduced from [8].

^b ^ Statements used for tailored assessment.

^c ^Items were reverse coded.

### Tailored Content

The steps involved in the production and presentation of tailored messages [22,25] are described subsequently.

#### Developing Tailoring Assessment Feedback Statements

Statements upon which tailored feedback would be based were identified. To maximize message salience and minimize message length, the item from each of the PHM factors that loaded most highly on that factor provided the message utilized [7] because previous research has established the cross-national validity of the PHM [10]. A “cancer worries” statement was not included because concern was addressed in statements derived for other variables. The fecal aversion and self-efficacy tailored feedback statements were chosen on the basis that they included aspects of all aversion or self-efficacy statements. The form of message feedback provided was determined by the strength of the rating provided by the participant. The aim was to reinforce the person when they provided feedback that was consistent with screening participation and to motivate those respondents whose ratings were inconsistent with screening participation. Progression of such messages is illustrated in Table 3; a similar series of messages was developed for each variable.

**Table 3:** Creating a library of tailored messages for the Preventive Health Model (PHM) factor “response efficacy” presented in order from reinforcing to motivating.

Factor	Response efficacy^a^
Tailoring statement	When colorectal polyps are found and removed, colorectal cancer can be prevented.
Strongly agree (5)	[Name], *you’ve told us that colon cancer screening is effective. You’re absolutely right. *That is why the Australian Cancer Council recommends yearly screening for people over 50 who are of average risk. It’s an important step to take to protect your health for the future, and could save your life.
Agree	[Name], *you’ve told us that you believe colon cancer screening is effective. You’re right. *That is why the Australian Cancer Council recommends yearly screening for people over 50 who are of average risk. It’s an important step to take to protect your health for the future, and could save your life.
Not sure	[Name], *you’re not sure that colon cancer screening is effective. It’s very effective*—that’s why the Australian Cancer Council recommends yearly screening for people over 50 who are of average risk. As you are [age], it’s an important step to take to protect your health for the future, and could save your life.
Disagree	[Name], *you don’t think that colon cancer screening is effective. In fact it’s very effective*—that’s why the Australian Cancer Council recommends yearly screening for people over 50 who are of average risk. As you are [age], screening could save your life by finding early, curable cancer.
Strongly disagree (1)	[Name], *you really don’t believe that colon cancer screening is effective. In fact it’s very effective*—that’s why the Australian Cancer Council recommends yearly screening for people over 50 who are of average risk. As you are [age], screening could save your life by finding early, curable cancer.

^a ^Tailoring “fragments” shown in italics. Personalized fields indicated in square brackets.

#### Developing Tailoring Decision Rules

Decision rules were developed for each tailored feedback variable based on “if-then-else” logic. Messages that addressed PHM factors shown from previous research to be predictive of moving people from their current decision stage to a “better” stage [14] were given priority in the presentation of feedback to individuals, to exploit the primacy effect [26]. The two factors most strongly related to each decision stage (presented first to those in that stage) are shown in Table 4.

**Table 4:** Relating Preventive Health Model (PHM) factors to Precaution Adoption Process Model (PAPM) decision stage for colorectal cancer screening via fecal occult blood test (FOBT).

PAPM decision stage	PHM factors most strongly associated with decision stage^a^
Never heard of FOBT	Salience and coherence, susceptibility
Not considered FOBT	Susceptibility, response efficacy
Decided against FOBT	Susceptibility, self-efficacy
Undecided about FOBT	Salience and coherence, self-efficacy
Decided to use FOBT	Response efficacy, self-efficacy

^a ^Source: [14].

#### Preparation and Presentation of Tailored Messages

**Figure 1:**
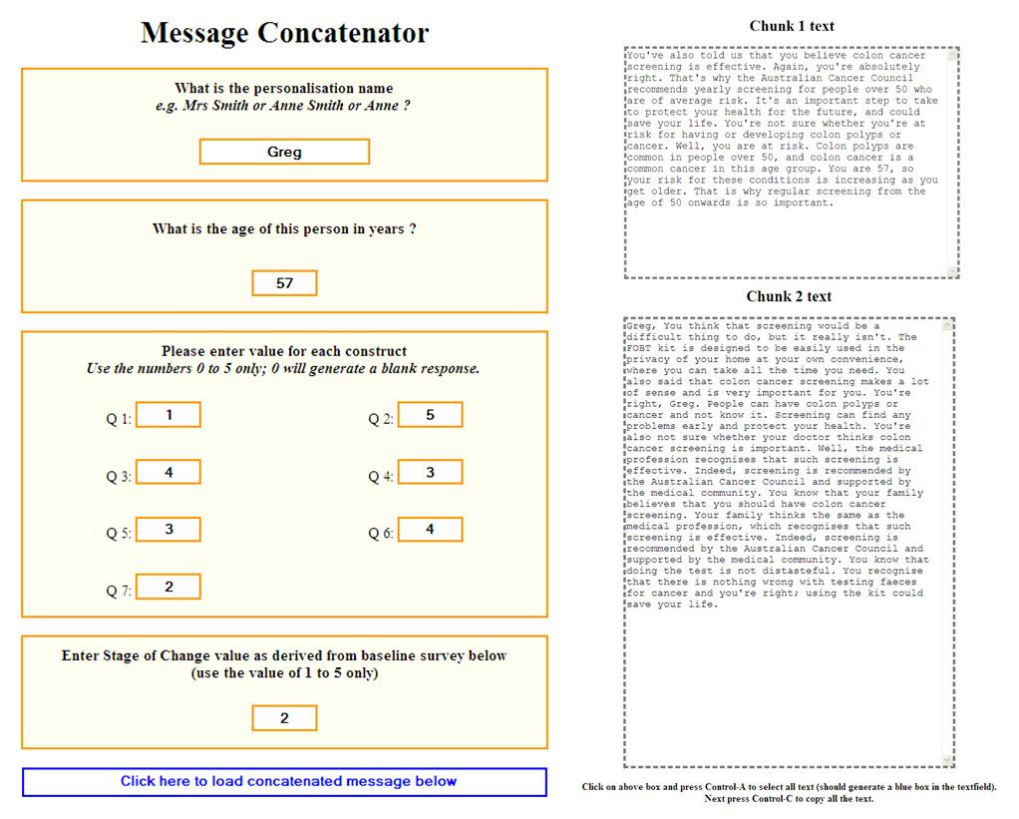
Message concatenator entry and resulting tailored message chunks.

A combination of programming and manual steps ensured correct presentation of messages. From the baseline survey data, an access query obtained the participant’s name, age, scores on each tailoring variable, and screening decision stage. This information was manually entered into a logic program, a “message concatenator” that accessed the message library and produced a set of personalized reinforcing or motivating messages according to scores. The messages were divided into “chunk 1” (messages addressing the factors most salient to the participant’s decision stage as seen in Table 4) and “chunk 2” (messages addressing the remaining factors as seen in Table 2). The text of both sets of messages varied by individual—those at the same decision stage (having the same salient and less salient factors relating to that decision stage) may have scored those factors differently, so messages would reflect the divergence in scores. An example of a completed concatenator form and the resultant set of messages is shown in Figure 1.

As Figure 1 indicates, the underlying message–merge syntax resulted in duplication of words and phrases, concepts running together, and seemingly random placement of the personalized name and age details. These messages were subsequently edited by the first author in order to omit duplicated phrases and enhance flow between PHM factor concepts. Following is an example of a portion of the final edited message format.

##### Chunk 1 Text, Your Information (Part 1)

Greg, you believe that colon cancer screening is effective. You’re absolutely right. That’s why the Australian Cancer Council recommends yearly screening for people over 50 who are of average risk. It’s an important step to take to protect your health for the future, and could save your life.

However, you’re not sure whether you’re at risk for having or developing colon polyps or cancer. Well, you are at risk. Colon polyps are common in people over 50, and colon cancer is a common cancer in this age group. You are 57, so your risk for these conditions is increasing as you get older.

##### Chunk 2 Text, Your Information (Part 2)

You think that screening would be a difficult thing to do, but it really isn’t. The FOBT kit is designed to be easily used in the privacy of your home at your own convenience, where you can take all the time you need.

You do think colon cancer screening makes a lot of sense. You’re right, Greg. People can have colon polyps or cancer and not know it. Screening can find any problems early and protect your health.

Once edited, for the Web-based/tailored group, messages were entered into a program that displayed them on the computer screen, matched to the participant for whom they were intended. Messages bound for the paper-based/tailored group were copied and pasted into the appropriate spot in the personalized materials presented to each participant.

### Generic Information and Educational Content

Generic information statements to encourage colorectal cancer screening are shown in Textbox 1.

Educational material was provided to all groups as a component of informed decision making [27]. Its content closely followed the format of information presented in the NBCSP consumer information booklet [28] and although it did not go into detail about the risks of screening (false negatives that can lead to people being wrongly assured and false positives that can result in unnecessary anxiety and diagnostic procedures), the material reproduced information that would be received by those targeted in the Australian population-based screening program. An indicative extract from the table of contents is shown in Textbox 2.

**Figure 2:**
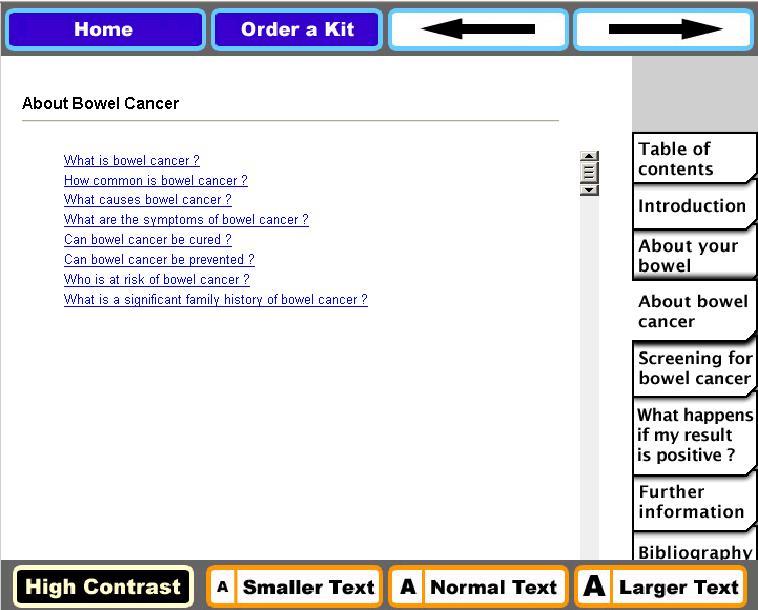
Screenshot of Web-based educational content.

The paper group received the educational material in the form of a series of pages arranged in typical paper-based fashion (ie, with a table of contents and corresponding page numbers) and text that progressed according to the table of contents. The Web group received the same material but as a series of headings, tabs, and hyperlinks. Web pages were designed to reflect issues of vision and cognition needs of older users; for example, the ability for text to be enlarged, contrast to be changed, and use of adequate white space [29]. A sample of the Web page is shown in Figure 2.

**Generic Information Statements to Encourage Colorectal Cancer Screening.**

Some important messages concerning screening for colorectal cancer:

- People can have colon polyps or cancer and not even know it. Colon polyps are common in people over 50, even in those with no family history. Finding colon cancer early and removing colon polyps when they are small can prevent cancer.
- The risk for these conditions increases with age; that’s why regular screening from the age of 50 onwards is so important.
- Colon cancer screening is very effective—that’s why the Australian Cancer Council recommends yearly screening for people over 50 who are of average risk.
- The medical profession recognizes that such screening is effective, and supports the Australian Cancer Council recommendation for yearly screening.
- Colorectal cancer screening is easy to do. The kit is designed to be quickly and easily used in the privacy of your home at your own convenience.
- Some people may think that doing the test might be unhygienic or embarrassing, but this needn’t be so. Recognizing that there is nothing wrong with testing feces for cancer and using the kit could save lives.

**Web- and Paper-Based Educational Content: Extract of Major Headings From the National Bowel Cancer Screening Program Consumer Information Material [28].**

About bowel cancer:

- What is bowel cancer?
- How common is bowel cancer?
- What causes bowel cancer?
- What are the symptoms of bowel cancer?
- Can bowel cancer be prevented?
- Who is at risk of bowel cancer?

Screening for bowel cancer:

- What is screening?
- What does the FOBT involve?

What happens if my result is positive?

- What does a colonoscopy involve?
- Are there any risks from a colonoscopy?

### Screening Offer

An FOBT order form accompanied the print and Web material. Participants had the option of completing the form immediately after viewing the material and leaving the completed form with the researchers (paper group) or using a hyperlink to email their details (Web group). The screening kit included (1) a bowel cancer screening information pamphlet; (2) an immunochemical FOBT (InSure, Enterix Australia) that does not require dietary or drug restrictions; (3) a combined participant details and consent form confirming personal details, nominating a preferred doctor for follow-up, and consent to obtain clinical follow-up reports if required; and (4) a reply-paid return envelope addressed to the processing laboratory. The processing laboratory provided the researchers with de-identified FOBT receipt information.

### Data Analysis

Chi-square analyses (χ^2^) and one-way analyses of variances (ANOVAs) were initially undertaken to confirm group comparability at baseline. A mixed-group design ANOVA with two between-group factors—message delivery modality (Web or paper) and message type (tailored or non-tailored)—and one within-subject factor, time of measurement (baseline–time 1 and endpoint–time 2), was undertaken to examine for main effects from message, modality, and time of measurement, and their interaction on the main outcome variables. These main effects and interaction terms were examined in order to test whether attitudes and beliefs, as reflected in scores on the PHM variables and self-efficacy and fecal aversion, improved over time, and were influenced by the message delivery modality and the nature of the message. It was hypothesized that tailored messages delivered via Web modality would be associated with greatest improvement. The effect of modality and message type on categorical outcome variables (ie, decision stage movement, FOBT request, and uptake) was examined using Chi-square analyses. Participation rates were defined as “early” or “late” at a cut-off point of 6 weeks following dispatch of FOBT, and “non-return” at a cut-off point of 12 weeks. All analyses were conducted using a two-sided alpha level of .05.

## Results

From a sampling frame of 756 people, 532 people were contacted at baseline. Of these, 298/532 (56.0%) declined to participate and 134/532 (25.2%) were ineligible. In total, 100/756 (13.2%) agreed to participate in the study. There was no significant difference in gender distribution (the only demographic variable obtained from non-participants) between those who declined to participate and participants (χ^2^_1 _= 0.2, *P *= .68). Because the numbers in the intervention groups were lower than anticipated, we resorted to “snowball” [30] recruitment (n = 19) to increase the number of participants to within approximately equal numbers in each group. “Snowballing” is a chain-referral method whereby a study participant who fits the eligibility criteria uses their social networks to recruit participants with similar characteristics. Those participants then recruit others, resulting in a process analogous to a snowball rolling down a hill. Subsequently, 15 participants (2 in the paper/non-tailored, 5 in the paper/tailored, 6 in the Web/non-tailored, and 2 in the Web/tailored groups) did not attend the laboratory for the intervention. Therefore, full data were available for 95.7% (104/119) of participants. Although “snowball” recruits comprised 17.3% (18/104) of the final sample and varied between groups, there were no significant differences between groups for gender, mean age, education, Australian birth, marital status, and awareness of FOBT (Table 5).

**Table 5:** Comparison of groups across conditions.

Demographic		Condition					χ^2^ (df)	*F* (df)	*P*
		Paper		Web		Control (n = 20)
		Non-tailored (n = 22^a^)	Tailored (n = 21^b^)	Non-tailored (n = 20^c^)	Tailored (n = 21^d^)				
**Gender, n (%)**							4.34 (4)		.36	
	Male	15 (68)	10 (48)	10 (50)	8 (38)	9 (45)			
	Female	7 (32)	11 (52)	10 (50)	13 (62)	11 (55)			
**Education level, n (%)**							8.53 (8)		.38	
	Some high school	6 (27.3)	7 (33.3)	4 (20)	7 (33.3)	10 (50)			
	Completed high school/trade	6 (27.3)	6 (28.6)	8 (40)	7 (33.3)	8 (40)			
	University	10 (45.4)	8 (38.1)	8 (40)	7 (33.3)	2 (10)			
**Place of birth, n (%)**							7.01 (4)		.13	
	Within Australia	18 (81.8)	15 (71.4)	13 (65)	18 (85.7)	19 (95)			
	Outside Australia	4 (18.2)	6 (28.6)	7 (35)	3 (14.3)	1 (5)			
**Relationship status, n (%)**							1.79 (4)		.77	
	With partner	18 (81.8)	15 (71.4)	17 (85)	15 (71.4)	15 (75)			
	Single	4 (18.2)	6 (28.6)	3 (15)	6 (28.6)	5 (25)			
**Heard of FOBT, n (%)**							2.32 (4)		.68	
	Never heard of FOBT	11 (50)	9 (42.9)	10 (50)	12 (57.1)	13 (65)			
	Heard of FOBT	11 (50%)	12 (57.1)	10 (50)	9 (42.9)	7 (35)			
Age, mean (SD)		61 (7.0)	62 (6.4)	60 (6.2)	59 (7.9)	62 (6.8)		0.75 (4.99)	.56

^a ^Snowball (n = 1).

^b ^Snowball (n = 6).

^c ^Snowball (n = 6).

^d ^Snowball (n = 5).

### Change in PHM Scores

Initial examination of movement in attitudes and beliefs of the control group across time were examined using related samples *t *tests. No significant changes were observed, suggesting that scores on PHM variables and fecal aversion and self-efficacy, without intervention, were all stable and reliable across time.

Descriptive statistics for intervention groups according to condition (ie, 2 × 2 × 2) are presented in Table 6, and the results of the repeated measures ANOVAs are presented in Table 7. Overall, there was a significant change in scores pre- and post-intervention for all variables. However, there were no significant time × modality interactions, indicating no difference between groups due to receiving paper versus Web-based information. There were significant time × message interactions for both perceived susceptibility and self-efficacy. Perceived susceptibility scores for individuals receiving tailored information increased from mean 10.6 (SD 2.1) to mean 11.8 (SD 2.2). Scores on self-efficacy increased in the tailored group from mean 11.7 (SD 2.0) to mean 12.6 (SD 1.8). There were significant time × modality × message effects for social influence and salience and coherence, reflecting an increase in these scores for tailored Web-based participants only: social influence scores increased from mean 11.7 (SD 2.6) to mean 14.9 (SD 2.3) and salience and coherence scores increased from mean 16.0 (SD 2.2) to mean 17.7 (SD 2.1).

**Table 6:** Means and standard deviations on all PHM, fecal aversion, and self-efficacy outcome variables according to condition.

Outcome variable	Message type	Pre-intervention modality		Post-intervention modality	
		Paper mean (SD)	Web mean (SD)	Paper mean (SD)	Web mean (SD)
Salience and coherence	Tailored	16.4 (2.5)	16.0 (2.2)	16.6 (2.3)	17.7 (2.1)
	Non-tailored	17.0 (2.3)	15.8 (2.4)	17.6 (1.8)	16.1 (2.5)
Cancer worries	Tailored	6.3 (1.6)	5.2 (2.1)	5.9 (1.9)	4.3 (2.1)
	Non-tailored	4.5 (2.1)	5.0 (1.9)	4.4 (1.8)	5.0 (1.9)
Perceived Susceptibility	Tailored	10.4 (2.1)	10.8 (2.1)	11.4 (2.1)	12.3 (2.3)
	Non-tailored	10.9 (1.6)	10.8 (2.3)	11.0 (2.2)	10.9 (2.1)
Response efficacy	Tailored	7.5 (1.1)	7.7 (1.1)	8.0 (1.2)	8.1 (1.1)
	Non-tailored	7.4 (1.0)	7.7 (1.3)	8.1 (1.3)	8.2 (1.0)
Social influence	Tailored	13.5 (1.9)	11.7 (2.6)	14.5 (2.0)	14.9 (2.3)
	Non-tailored	12.7 (2.7)	12.9 (2.6)	14.9 (2.5)	14.1 (2.9)
Self-efficacy	Tailored	11.7 (2.2)	11.7 (1.9)	12.1 (1.6)	13.0 (1.9)
	Non-tailored	12.5 (1.3)	11.9 (1.4)	12.4 (1.4)	12.2 (1.5)
Fecal aversion	Tailored	9.5 (2.5)	10.4 (3.0)	10.4 (2.6)	11.4 (2.8)
	Non-tailored	11.2 (2.0)	10.3 (2.1)	11.9 (2.2)	10.6 (2.7)

**Table 7:** Repeated measures ANOVAs comparing pre- and post-intervention group scores.

Outcome variable	Time^a^		Time × Modality		Time × Message		Time × Modality × Message	
	*F*_1,80_^b^	*P*	*F*_1,80_^b^	*P*	*F*_1,80_^b^	*P*	*F*_1,80_^b^	*P*
Salience and coherence	10.28	*< .001*	1.62	.20	1.04	.31	4.25	*.04*
Cancer worries	5.86	*.02*	0.28	.59	2.90	.09	0.88	.35
Perceived susceptibility	11.15	*< .001*	0.27	.60	7.11	*.01*	0.52	.47
Response efficacy	14.49	*< .001*	0.15	.69	0.41	.52	0.31	.57
Social influence	65.80	*< .001*	1.54	.22	0.50	.48	11.03	*< .001*
Self-efficacy	8.62	*< .001*	3.21	.08	4.74	*.03*	0.58	.45
Fecal aversion	16.60	*< .001*	0.15	.70	1.27	.26	0.42	.52

^a ^Time effect refers to pre- and post-intervention scores.

^b^ *F *test for statistical difference between > 2 groups.

### Movement in PAPM Decision Stage

**Figure 3:**
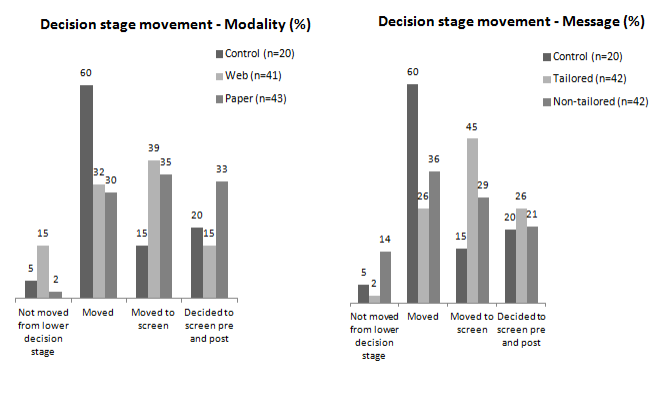
Movement in decision stage post intervention.

Movement in the PAPM decision stage from pre- to post-intervention was measured for modality and message separately, and included the control group (Figure 3, shown as a percentage of group). A greater percentage of the control group “moved” compared to intervention groups; however, they moved from “unaware of the issue” to only “heard of the issue but unconcerned.” Although a similar percentage of Web- or paper-based participants moved to deciding to screen, a greater percentage of those receiving tailored (vs non-tailored) messages moved to deciding to screen. Post-intervention movement of intervention groups was further dichotomized as “moved to screen” versus “other movement type” and excluded those who had decided to screen pre- and post-intervention (n = 20). A Chi-square (with Yates continuity correction) analysis indicated no significant difference for either modality (χ^2^_1 _= 0.2, *P *= .62) or message (χ^2^_1 _= 2.3, *P *= .13), indicating that Web or tailored delivery was no more effective than paper or non-tailored delivery in moving people toward a decision to screen.

### FOBT Participation

Including the control group, FOBTs were requested by 58.7% (61/104) of participants at the interview and by another 9 participants following the interview (overall request rate 67.3%, 70/104). Completed FOBTs were returned by 58.6% (41/70) of participants over a period of 14.1 weeks (mean 4.9 weeks). Of the kits returned, 65.9% (27/41) were received within 6 weeks (“early”). For the intervention groups, there was no significant difference in modality (Web vs paper) or message (tailored vs non-tailored) for FOBTs requested or returned (Table 8).

**Table 8:** Request and return of fecal occult blood tests (FOBTs) by intervention group.

	Modality				Message				Control
	Web n = 41	Paper n = 43	χ^2^_1_	*P*	Tailored n = 42	Non-tailored n = 42	χ^2^_1_	*P*	n = 20
FOBTs requested	28	34	0.8	.38	34	28	1.5	.21	8
FOBTs returned	18	18	0.0	.99	22	14	2.4	.12	5

### Participant Interviews

Interviews were conducted with all intervention group participants by IF and EH, in groups or alone according to number of participants at each session. They discussed issues relating to acceptability of the information, particularly the tailored messages, and acceptability of the website.

#### Impact of Tailored Message

Respondents who received tailored information had a positive impression overall. The tailored information was viewed as an acceptable substitute for a one-on-one conversation:

male, Web/tailored group It was really in response to the information I had given, and, it wasn’t patronizing but it was really just confirming...Yes, I think that approach is beneficial. It drags you in; it’s like having a conversation.

The fact that the tailored information addressed an individual’s specific responses was positively regarded:

female, Web/tailored group I think it reinforced things for me...I’d made one comment that I perhaps agreed or disagreed, but this was actually telling me why it was different...So for me it’s a more direct approach and I found that very useful.

Although some participants did experience the receipt of tailored information as somewhat confronting:

female, paper/tailored group I sort of didn’t realize what I had said. Well, I think probably I’ve thought about it since, and thought well that was a stupid statement to make...it was just a bit strange seeing things that I’d said.

#### Acceptability of the Website

The presentation of the Web tool was described as requiring improvement:

*I’d be looking for clear indication on the site that gave me very quickly, very clearly without a lot of words, what I’ve gotta *[sic] *look for and what I can do to find out. It was a bit long and a bit wordy—trying to put too much information. You can do more with pictures than you can do with words. *[male, Web/non-tailored group]

female, Web/non-tailored group ...it wasn’t very exciting. It was very boring...there weren’t any pretty pictures, it was sort of, you know—very basic.

male, Web/non-tailored group I think beige would be more exciting!

#### Impact of Educational Content

Some male participants were surprised to learn that CRC and prostate cancer incidence rates are similar. This suggested some men were more aware of prostate cancer screening and considered themselves more at risk for prostate cancer than CRC:

*It rose *[sic] *my awareness. I’m now aware that that’s a test that I should be looking for. And put it in the same bracket as prostate cancer, whereas it was not even on my radar. *[male, paper/tailored group]

*When I saw the graph and I saw prostrate *[sic] *cancer and I saw bowel cancer and they were almost identical, can’t remember the exact numbers, but they were almost identical in terms of amount. *[male, Web/tailored group]

## Discussion

This exploratory study tested the relative efficacy of Web-delivered, tailored messages about CRC screening and FOBT use on beliefs about and attitudes toward screening in comparison to paper-delivered tailored messages and non-tailored messages delivered by both paper and Web. In addition to changes in PHM variable scores, outcomes included changes in decision stage for screening, FOBT requests, and participation.

After the interventions, there was an improvement in PHM variable scores for all groups except the control group, who received no information. For the intervention groups, all mean scores significantly moved in the desired direction (eg, decreased cancer worries or increased support for cancer screening identified in all other psychological variables). Although not influencing every factor, receipt of tailored messages increased perceived susceptibility and self-efficacy and increased both salience and coherence and social influence when combined with Web delivery. Perceived susceptibility has been shown to be a predictor of intention to screen [31,32]. Similarly, a person’s confidence in their capacity to act (self-efficacy) is widely reported as a predictor of actual health behavior participation [33] and has been shown to moderate the relationship between intention, planning, and action [34,35]. Thus, it appears that messages tailored to individual levels of these important factors have a greater likelihood of beneficially influencing screening behavior than more generic messages. Salience and coherence and social influence are also important behavioral determinants of screening [8]; it is unclear how Web delivery interacted with tailoring to improve these scores.

Overall, there was no indication of a modality effect for PHM factors; the delivery channel alone (paper or Web) had no direct influence on score changes. Web delivery enables a shift from the use of static material to a dynamic interactive resource [36] that could be expected to provide more sophisticated and effective decision support. Qualitative data suggests that the site may not have fulfilled expectations with regard to “media richness.” “Rich” media are generally characterized by the capacity for immediate feedback, the capacity to transmit multiple cues, the use of language variety, and capacity of the medium to have a personal focus [37,38]. The Web-based educational content of the decision support tool was designed to be comparable with the paper version, with only the presence of hyperlinks differentiating the modes of transmission (Figure 2). Interview feedback suggested participants found the information “boring” and “dry” in comparison to other websites and this may have affected their level of engagement. This result highlights the need to ensure that Web-based information is presented in media-rich format that users have come to expect, albeit with due consideration of the needs of the target age group (eg, issues of cognition, readability, vision, and disability) [29].

Regarding movement in decision stage, more people in the control group moved from “never heard of FOBT” to no further than “not considering” FOBT. This result corresponds with the control group’s lack of movement in PHM scores and clarifies that the act of being asked to just think about the factors associated with screening without accompanying more specific information is unlikely to encourage screening uptake. Although there was greater movement toward a decision to screen in the intervention groups, no one modality or message type was more effective than the other. The lack of effect of Web versus paper delivery could be ascribed to the previously mentioned lack of expected Web-based richness of information. However, receipt of tailored messages, compared to non-tailored material, had a beneficial effect on several PHM factors and could be expected to increase intention to screen.

Despite a growing evidence base showing that tailored messages are superior to generic messages in their ability to influence health behavior [39], the mechanisms by which tailoring works is still unclear [40]. Tailored feedback can take different forms—descriptive, comparative, evaluative, or a combination [25,41]—and their relative effectiveness may differ between individuals. For example, some study participants found our descriptive approach (providing feedback on what is known about the recipient based upon their PHM responses) confronting (eg, it was a “...bit strange seeing things that I’d said”) and the language may have lacked empathy. Message framing may also have an influence. For example, Akl and colleagues [42] in their systematic review noted that loss messages (vs gain messages) led to more positive perception of effectiveness for screening messages. Others have found that differing presentation of risk factors (absolute vs comparative) had an impact on intention to screen [43], and message order was found to influence responses to breast cancer information [44]. Further research to test the effects of different types of message feedback, framing, and presentation order using both behavioral and communication theories [45,46] in the CRC screening context would help “unpack” the mechanisms through which tailoring has an influence.

The FOBT request and uptake rates, although greater than the control, were no different for modality or message type. Only slightly more than half of the FOBTs requested were returned. Although it is generally accepted that intention to screen is a necessary precursor of action, other variables amenable to tailoring may exert a greater influence on screening uptake. For example, in a group of people committed to screening we found that, in conjunction with self-efficacy, commitment explained only 8.0% of variance [47], and others have found that life difficulty variables were better predictors of action than intention [48]. Other researchers have approached the choice of tailoring variables in other ways (eg, by targeting the most important barriers identified by participants themselves [49]).

We did not test whether knowledge of bowel cancer and screening was enhanced through provision of the educational material, or whether such knowledge helped participants decide whether or not to screen. Knowledge is a critical component of informed decision making; however, as Jepson and colleagues [50] point out, a tension exists between the need of a screening program to attain high rates of uptake and the promotion of informed choice—an individual with whom information about the explicit risks and benefits has been shared may choose not to undertake screening (as in the case of prostate cancer screening [51]). Although acknowledging the ethical imperative of being able to make an informed choice, it is unclear whether increased knowledge alone actually influences uptake. Increased knowledge does not necessarily translate into action. This fact has been demonstrated with respect to bowel cancer screening [52,53], other screening behaviors [50,54-56], and organ donation [57].

Regardless of the relative lack of effect of Web-based delivery, from a practical perspective, using the Web as a delivery medium for tailored information has significant advantages over tailoring via paper. Material and decision rules can be created and updated more easily and economically, thus maintaining currency for a longer period, and the interaction required for obtaining relevant information and providing tailored feedback can occur in the one session. This creates a “real time” interaction that can be linked to immediate behavior activation (eg, ordering an FOBT online). Older people are increasingly using the Internet. An earlier study we conducted found that more than half the population over 50 years in South Australia had access to the Internet at some location [58]. Others have found that in South Australia the proportion of people aged 45 to over 65 years seeking Internet health information significantly increased between 2001 and 2008 [59]. These data are likely to be representative of greater Australia and other developed countries.

### Limitations and Strengths

There are some limitations with this study. First, our sample was small, but it was consistent with that typically used in exploratory studies. Second, those who did participate were likely to have had greater focus on their health status and, therefore, not necessarily representative of the external population. Participants and non-participants did not differ in gender, although we acknowledge that they may have differed on other variables. It was not possible to measure the extent of any potential bias without detailed information on those who did not participate, which was inherently unavailable. We also had a lower than expected initial uptake rate, necessitating snowball recruitment and the associated loss of randomization.

Nevertheless, by means of this exploratory study we gained sufficient indication of the beneficial effect of tailored material on FOBT screening attitudes and participation to justify the formulation of feasible hypotheses upon which to expand our research. We also gained a participant perspective of the usability and the content of the website. These results will be incorporated into the design of a larger, truly randomized trial using an improved Web interface. The process of producing and presenting tailored messages from survey responses was labor-intensive and not readily transferable to population settings. Going through the process, however, highlighted the potential for the development of more sophisticated, fully automated message libraries and the use of natural language generation systems, for example [60,61].

Despite that randomization was broken, strengths of the study included the majority of participants (although self-selected) were randomly sampled from a population frame, thereby providing a stronger indication of generalizability of results. We used behavioral constructs that had been validated as predictors of CRC screening, and FOBT participation was not measured by self-report alone.

### Conclusions

This exploratory study has confirmed that the provision of tailored messages that address attitudes and perceptions of screening that are amenable to change are more likely to result in increased readiness to screen for CRC compared to provision of generic information alone. However, despite increased PHM scores and generally positive qualitative feedback, tailored messages did not result in significantly increased requests for an FOBT or its actual use. Future research should address optimal message framing and construction, and consideration of other possible psychosocial influences on screening uptake. Mode of delivery did not affect outcomes, but this may have been due to Web design deficiencies. From a public health promotion perspective, the Web has economic and logistical advantages over paper as a delivery medium.

We are currently undertaking a large-scale, randomized population trial using a redesigned Internet decision aid [62] to construct and deliver tailored messages in real time. Based on the results of this exploratory study, we have been able to improve the quality and precision of our intervention. We ensured that the initial sample approached would be large enough, after allowing for attrition through non-eligibility and non-participation, to retain sufficient power to detect statistically significant group differences for the primary outcomes. This study also highlighted that the steps involved in gaining responses to PHM variables upon which to base tailored messages, and the process of preparing the messages for presentation in a coherent manner, was labor-intensive and not compatible with a population-based screening program. Therefore, in the larger trial [62], baseline survey responses will be collected in real time and participants in the tailored information group will receive immediate tailored feedback. Additionally, an automated tailored message library using sophisticated algorithms [61] will be used to ensure that messages are united with natural language so that they can be read in a coherent, logical manner without the need for further “editing.”

## Abbreviations

ANOVA: analysis of variance
CATI: computer-assisted telephone interview
CRC: colorectal cancer
CSIRO: Commonwealth Scientific and Industrial Research Organisation
FIT: fecal immunochemical test
FOBT: fecal occult blood test
iFOBT: immunochemical fecal occult blood test
NBCSP: National Bowel Cancer Screening Program
PAPM: Precaution Adoption Process Model
PHM: Preventive Health Model
